# Descriptive epidemiology of prenatal and perinatal risk factors in a Chinese population with reading disorder

**DOI:** 10.1038/srep36697

**Published:** 2016-11-07

**Authors:** Lingfei Liu, Jia Wang, Shanshan Shao, Xiu Luo, Rui Kong, Xiaohui Zhang, Ranran Song

**Affiliations:** 1 Department of Maternal and Child Health and MOE (Ministry of Education) Key Laboratory of Environment and Health, School of Public Health, Tongji Medical College, Huazhong University of Science and Technology, Wuhan, 430030, China

## Abstract

Several prenatal and perinatal factors have been found to be associated with developmental dyslexia (reading disorder) in alphabetic language. Given the absence of relevant studies of Chinese children, the present study tries to investigate these risk factors. A total of 45,850 students were recruited from grades three to six, from seven cities of Hubei province. Dyslexia in Chinese was diagnosed based on children’s clinical symptoms. The clinical symptoms of children’s reading performance were assessed by Dyslexia Checklist for Chinese Children (DCCC) and Pupil Rating Scale Revised Screening for Learning Disabilities (PRS) which were completed by parent/guardian and header teacher respectively. Chinese language exam was used to screen children with poor reading capacity. Questionnaires about prenatal and perinatal factors were completed by parent or guardian. Among the 34,748 eligible participants, 1,200 (3.45%) were diagnosed with dyslexia in Chinese. More boys suffered from dyslexia than the girls and the gender ratio was 3:1. Family history of neuropsychiatric diseases, maternal infectious diseases, difficult vaginal delivery, preterm birth, and neonatal asphyxia were found to increase the risk of developmental dyslexia in China. Closer longitudinal developmental monitoring and preventive measures should be taken for high risk children.

Developmental dyslexia is a disorder in children with normal intelligence and sensory abilities who show specific reading difficulties in accurate and/or fluent word recognition, spelling, and decoding abilities across different languages^1^. Children who suffer from dyslexia have more depressive moods, anxiety symptoms, somatic complaints, and behavioural problems than their peers who are normal readers^2^,^3^,^4^,^5^. A majority of children with dyslexia have persistent disorders in reading, which therefore impairs education attainment and income in adulthood^6^.

The prevalence of dyslexia varies across different languages and countries. For alphabetic languages, dyslexia is fairly pervasive with a prevalence ranging from 5% to 17.5%^7^. It was not until 1980s that the researchers realized the existence of Chinese dyslexia. A cross-culture study conducted in Taiwan, Japan and America in 1982 revealed that the prevalence of dyslexia in Chinese (7.5%) were similar with that in English (6.3%)^8^, and these bring more attention on Chinese dyslexia. A survey conducted in the academic year 1999/2000 in Hong Kong found that the rate of dyslexia was approximately 9.7% among students in grades one to four^9^. However, large-scale epidemiological study of dyslexia has been lacking in Mainland China in recent years.

Phonological processing is considered the core deficit of impaired reading in alphabetic and logographic language. A study in Chinese students found that children with impaired English reading have poorer Chinese reading performance than typical readers; functional magnetic resonance imaging results revealed that neural deficits involved for impaired phonological processing in English as the second language are similar with English as the first language^10^.

Evidences showed that dyslexia is associated with multiple genes and environmental risk factors. Nine risk loci (DYX1–DYX9) and six candidate genes (DYX1C1, DCDC2, KIAA0319, C2Orf3, MRPL19, ROBO1)^1^ are found to be linked with dyslexia. Besides, with the recent advances in genome-wide association studies^11^,^12^, more genes are found that may be involved in developmental dyslexia^13^,^14^. Parental education is an important environmental risk factor interacting with genes influence on reading disability^15^. Other possible environmental factors include the language and pre-literacy environments that parents provide to their children^16^. Our pervious screening study in a middle-size city found that mother’s education level, the time spend on electronic devices, and the literacy-related activity were associated with developmental dyslexia^17^,^18^. The purpose of this study was to explore potential environment risk factors for dyslexia from another aspect.

As is known, developmental dyslexia is a kind of neurodevelopmental disorder. Risk factors such as maternal malnutrition, drugs and alcohol, maternal diseases, and unfavourable events during the perinatal period could disrupt normal brain development and lead to dysfunction. Several factors have been reported to have adverse effects on reading or language ability. Preterm birth was one of the most frequently reported risk factors on children’s reading disabilities. A recent meta-analysis found that school-aged children born preterm had poorer decoding and reading comprehension performance than their full-term peers^19^. A population-based cohort study indicated that former late preterm may not increase the risk of learning disabilities^20^. Study on school-aged children from Avon Longitudinal Study of Parents and Children (ALSPAC) found that prenatal nicotine exposure was associated with poor performance in specific reading skill outcomes, especially in decoding single words^21^. Mascheretti *et al.*^22^ reported that maternal smoking during pregnancy interacted with dyslexia candidate gene DYX1C1 and affected dyslexia-related reading, spelling and memory phenotypes. Maternal hypertension diseases were found reduced the verbal ability, not non-verbal ability, in the 10 aged children from Western Australian Pregnancy Cohort (Raine) Study^23^. van Handel *et al.*^24^ reviewed that mild neonatal encephalopathy following perinatal asphyxia increased the risk of reading and spelling difficulties in children with norm intelligence quotient. Children who affected neonatal hyperbilirubinemia had persisting problems with reading, writing and mathematics in a prospective birth cohort^25^.

The long-term effects of prenatal and perinatal risk factors on children’s neurodevelopment could be alleviated by effective intervention policies and therefore these differ in different countries and cultures. Conclusions draw from populations in alphabetic language environment may not be as applicable to Chinese children because of the different socioeconomic level and language backgrounds. Due to the lack of related studies in China, we conducted this descriptive epidemiological study of primary school students. Our aim was to screen prenatal and perinatal factors and elucidate the distribution of dyslexia in Chinese.

## Results

### The Prevalence and Distribution of Dyslexic Children

Of all the 34,748 students, 1,200 (3.45%) were diagnosed with dyslexia in Chinese. The prevalence of dyslexia varies in different cities (*P* = 0.02). The differences between the grades were significant (*P* < 0.001). There was a downward trend between dyslexia and grades (*P* for trend < 0.05), with the lowest prevalence (2.86%) among sixth grade students. More boys suffered from dyslexia than girls and the gender ratio was 3:1. The dyslexic children and non-dyslexic children were balanced on age (*P* = 0.316). See details in Table 1.

### Association between Prenatal and Perinatal Factors and Dyslexia

Table 2 presents the association between potential prenatal and perinatal risk factors and dyslexia, adjusted for maternal occupation, maternal education, and family income. The distribution of dyslexic children was different from non-dyslexic children with respect to maternal occupation, maternal education, and family income (*P* < 0.001). Dyslexic children had significant higher proportion of family history of neuropsychiatric diseases than non-dyslexic children (2.3% vs. 0.8%). We observed that the dyslexic children were significantly more likely than non-dyslexic children to have maternal infectious diseases (9.17% vs. 5.83%), maternal anti-infectious medication (2.18% vs. 1.39%), maternal antipsychotics (0.91% vs. 0.34%), and maternal smoking (0.53% vs. 0.20%) during prenatal period. The distribution of dyslexic children was different from non-dyslexic children on gestational weeks (*P* < 0.001). With regard to difficult vaginal delivery and neonatal asphyxia, the proportions of dyslexic children were higher than non-dyslexic children (3.12% vs. 1.75%, 3.76% vs. 1.42%).

After adjustment for possible confounding variables, the risk of dyslexia remained significantly associated with family history of neuropsychiatric diseases (*OR* = 2.15, 95% CI = 1.30–3.55), maternal infectious diseases (*OR* = 1.59, 95% CI = 1.25–2.03), preterm birth (*OR* = 1.30, 95% CI = 1.01–1.66), difficult vaginal delivery (*OR* = 1.58, 95% CI = 1.03–2.42), and neonatal asphyxia (*OR* = 2.38, 95% CI = 1.61–3.52).

## Discussion

In the present study, we observed that the prevalence of Chinese language developmental dyslexia was 3.45%. More boys suffered from dyslexia than girls. Family history of neuropsychiatric diseases, infectious diseases, difficult vaginal delivery, preterm birth, and neonatal asphyxia increased the risk of developmental dyslexia. As one of the rare large-scale surveys on Chinese dyslexia, our results not only demonstrate the prevalence and relative risk factors of dyslexia in Chinese, but also provide a data source for cross-culture study.

The prevalence obtained in our study is consistent with our previous result of 3.9% in a middle sample size research^17^. The result of our study is different from those obtained from Hong Kong (9.7%)^9^ and Taiwan (7.5%)^8^. The difference in prevalence between our study and the above studies may result from a variety of definition criteria. The diagnostic criteria of dyslexia applied in Hong Kong and Taiwan using the cut-off score of 1 SD below the mean. Our study used the cut-off of 2 SD from the mean. Types of script may play a role in determining the phenotype of dyslexia in Chinese students. As is known, simplified characters used in Mainland China are much easier to learn than traditional characters used in Hong Kong and Taiwan. An alphabetic phonetic system called *pinyin* used by Mainland Chinese students to read Chinese characters may also affect the prevalence of dyslexia.

There is a significant male predominance of dyslexia in our study (3:1) which is in the range of previously reported results (from 1.5:1 to 3.1:1)^26^. Compared with clinical data, our results to some extent avoid the bias result from high comorbidities in boys. Structural neuroimaging studies found less grey matter volume in the left inferior parietal cortex of dyslexic boys, while anatomical variants exist in early sensory and motor cortices of dyslexic girls^27^. Slower neural responses to speech of male adults compared with females helps to explain the higher incidence of reading impairment in males^28^. The mechanisms of sex differences in the structure and function of the brain and their role in dyslexia still needs more research.

It is known that dyslexia are familial and moderately heritable. Our results showed an association between family history of neuropsychiatric diseases and dyslexia. Shared genetic risk factors that influence the diseases may be the biologic mechanism underlying the link. Nucleotide changes in the ASD candidate gene, AUTS2, were reported to be associated with ADHD, epilepsy, and dyslexia^29^. However, conclusions about each specific neuropsychiatric disease could not be drawn from the present results and further investigation is needed.

We found an association between maternal infectious diseases and dyslexia. Epidemiological studies have shown that maternal infection could increase the risk of mental illness in offspring^30^. The mechanism for this probably involves the maternal immune response which may have an effect on the foetus. Maternal immune activation (MIA) resulting from prenatal exposure to infectious pathogens or inflammatory stimuli is increasingly recognised as playing an important etiological role in neuropsychiatric disorders with neurodevelopmental features^31^. The Interleukin-17a pathway, which is involved in the MIA of the virally infected mother, has been reported to induce behavioural abnormalities in offspring in rodent testing^32^.

There have been many improvements in perinatal care, resulting in the survival of a large number of infants with poor birth conditions, such as prematurity, birth disease, and abnormal delivery. Infants with poor birth conditions have greater risk of medical disabilities and behavioural and psychological problems in childhood than their peers. Our study showed increasing prevalence of dyslexia in students between grade three and grade six who had preterm births. Conclusions about the long-term effect of late preterm birth (34 to 37 weeks) on neurodevelopment, learning, and behavioural problems are inconsistent^20^,^33^. In our study, we did not have precise gestational weeks. Since most preterm births occur at moderate rather than extreme prematurity, it can be deduced that our results reflect the adverse effects of moderate preterm birth on reading ability. A magnetic resonance imaging study found that moderate and late preterm infants exhibit widespread brain white matter microstructural alterations compared with full-term controls^34^. Reduced organisation or volume of brain white matter in the left superior longitudinal fasciculus, which connects frontal and temporal language regions, may underpin developmental dyslexia^35^.

Neonatal asphyxia is the leading specific cause of neonatal mortality in low- and low-middle-income countries and is also the main cause of long-term morbidity including mental retardation, cerebral palsy, and other neurodevelopmental disorders^36^. Hypoxic ischemic brain injury is the main cause of neurodevelopmental sequelae in birth asphyxia survivors. The rodent model showed moderate hypoxia ischemia could reduce cortical activity, dendrite development, and impaired visual cortical plasticity^37^. Most studies about the long-term outcomes of infants with neonatal asphyxia are concentrated on the first two years; however, our study showed an association between asphyxia at birth and dyslexia at school age. The students we investigated are eligible to study in normal primary school without mental retardation and severe medical disabilities. It could be concluded that infants who survive neonatal asphyxia, even if they have no symptoms of neurological disorders, have a substantially increased risk of dyslexia.

After adjusting for neonatal asphyxia, difficult vaginal delivery is an independent risk factor for dyslexia. Prolonged duration of the second stage of labour could increase the risk of perinatal adverse outcomes^38^. An early follow-up study got contrary results, abnormal labour were not found associated with neurologic abnormalities (including speech delay and learning disabilities)^39^. Our data showed a mild but significant association between difficult vaginal delivery and child dyslexia, which suggested subtle neuronal damage. Since the cause, manifestation, and treatment of difficult vaginal delivery are complicated, consequently interpretation of its long-term effect on neurodevelopment should be done with caution.

Our study is an observational study about the prenatal and perinatal risk factors on dyslexia in Chinese, and prevalence and distribution were also described. We screened some risk factors, such as family history of neuropsychiatric diseases, maternal infectious disease, difficult vaginal delivery, preterm birth, and neonatal asphyxia.

There are several potential limitations to this study. Fist, this is a retrospective observational study and recall bias was unavoidable. We did not get objective information of prenatal and perinatal risk factors from medical records. Some detailed information such as specific medication, the severity of maternal disease, and the degrees of prematurity or asphyxia could not be obtained. However, we did receive relatively correct answers about some critical events, for example, whether the mother or infants had or had not suffered from particular diseases, since pregnancy and childbirth are special experiences for a family. Second, the participants in our investigation are from Hubei province, which may limit generalization to other Chinese population. This study provided clues for further study and data source for comparison with similar studies. Third, the present study had restricted power to detect the association between maternal smoking and antipsychotics and dyslexia. One possible cause is that the rates of maternal smoking and antipsychotics among pregnancy women are rare in China. This does not mean that children being exposed to smoking and antipsychotics in utero are risk-free of dyslexia, as it has been shown that maternal smoking and antipsychotics had adverse effect on offspring’s reading ability^21^,^40^. Finally, we did not obtain the family history of dyslexia and other language disorders, because our team was the first one studying dyslexia in Chinese in Hubei province. In summary, our study is a screen study and give a profile of prenatal and perinatal risk factors of dyslexia. Further investigation is needed to understand whether the observed associations are causal.

The findings from our study suggest that infants who are born with prenatal and perinatal risk factors, even if they are eligible to study in normal primary schools, would have higher risk of reading difficulty than their peers. It is necessary to undertake closer longitudinal developmental monitoring and preventive measures for high risk children.

## Methods

### Sources of Data and Ethics Statement

The data in this study were collected from an on-going project of the Tongji Reading Environment and Dyslexia Study (READ). The READ project launched in 2011 and aims to explore environmental and genetic factors affecting dyslexia. The READ was approved by the Ethical Committee of the Medical Association of Tongji Medical College, Huazhong University of Science and Technology. The methods of the present study were carried out in accordance with the approved guidelines. Written informed consent was obtained from the parents or guardians of the participant children after the nature of the study were explained.

### Participants

The present study was conducted on primary school students in seven cities of the Hubei province, including Qianjiang, Tianmen, Shayang, Suizhou, Jingshan, Yidu, and Yiling. The children enrolled in our investigation had passed the entrance examination and were eligible to study in normal primary school. Children with abnormal intelligence would recorded in school and were excluded from our study. In total, 45,850 students from grade three to grade six in selected schools participated in the investigation.

### Measuring tools

Questionnaires about prenatal and perinatal factors were filled out by parents or guardians. Instruction for filling and the contact information of our team were presented on the questionnaires, in case the fillers have any problems. The questionnaires also contain the variables of family socioeconomic status and home literacy environment. The Dyslexia Checklist for Chinese Children (DCCC) and Pupil Rating Scale Revised Screening for Learning Disabilities (PRS) were used to assess children’s daily reading behaviours and were completed by parent/guardian and header teacher respectively. Score of Chinese language exam in school was used to screen children with poor reading capacity.

The DCCC is a specialized rating scale for dyslexia in Chinese. The scale contains 58 items which were drawn from the clinical symptoms described in ICD-10, DSM-IV, and relative references. The responses are in a 5-point Likert-scale format ranging from ‘never do’ to ‘always do’ (1 = never do, 5 = always do). Higher score means more serious reading difficulty. The reliability coefficient is 0.724 (Cronbach’s a)^17^. The PRS is a convenient tool widely used to undertake a diagnose of learning disability in China, containing a total of 24 items representing 5 functional regions (i.e., listening comprehension, time and spatial judgments, social behaviour, motion ability, memory and language ability). The reliability coefficients (Cronbach’s a) were higher than 0. 90 for four functional regions and 0.84 for one region^41^. The score of each question ranges from 1 to 5. Higher score means better learning ability.

For the purpose of this study, dyslexia in Chinese was diagnosed using the following criteria: (a) the score of DCCC was 2 standard deviations higher than the mean score of all the students in same grade; (b) the score of PRS was lower than 65 points; (c) the Chinese language exam was below the tenth percentile of all children in the same grade; and (d) children who had suffered from intellectual disability, brain injury, visual and auditory disorders, epilepsy, and other neurological disorders were excluded.

### Data Collection

The number of questionnaires obtained from parents or guardians was 37,309 and 1,736 were excluded from analysis because of poor quality (missing rate more than 50%). In total, there were 35,573 valid parent/guardian questionnaires retained for further analysis. Among all the valid data, 5063 participants were from Qianjiang, which were reported in our pervious article^17^,^18^.

After excluding the data of children who had suffered from brain injury, visual and auditory disorders, epilepsy, and other neurological disorders, 34,748 were included in the final analysis.

### Statistical Analysis

Continuous variables were described as mean ± standard deviation (SD) and tested with *t*-test; Categorical variables were described as proportion (%) and tested with Chi-square test or Fisher’s exact probability. Logistic regression analysis was performed to calculate the odds ratio (*OR*) and 95% confidence intervals (95% CI) of the association between dyslexia in Chinese and prenatal and perinatal risk factors. All *P* values were two-tailed with a significant level of 0.05. Statistical analyses were carried out using SPSS 13.0 software.

## Additional Information

**How to cite this article**: Liu, L. *et al.* Descriptive epidemiology of prenatal and perinatal risk factors in a Chinese population with reading disorder. *Sci. Rep.* **6**, 36697; doi: 10.1038/srep36697 (2016).

**Publisher’s note**: Springer Nature remains neutral with regard to jurisdictional claims in published maps and institutional affiliations.

## Figures and Tables

**Table 1:** The prevalence and distribution of dyslexic students (n = 34,748).

	Non-dyslexic n (%)	Dyslexic n (%)	Chi-square/*t* value	*P* value
City			15.65	0.020
Qianjiang	4868 (96.15)	195 (3.85)		
Tianmen	6085 (96.33)	232 (3.67)		
Shayang	4031 (96.53)	145 (3.47)		
Suizhou	3750 (96.77)	125 (3.23)		
Jingshan	3800 (96.94)	120 (3.06)		
Yidu	6715 (96.22)	264 (3.78)		
Yiling	4299 (97.31)	119 (2.69)		
District			0.01	0.990
non-urban	12042 (96.54)	431 (3.46)		
urban	21506 (96.55)	769 (3.45)		
grade			16.92	<0.001
3 grade	7901 (96.15)	316 (3.85)		
4 grade	8387 (96.19)	332 (3.81)		
5 grade	8591 (96.66)	297 (3.34)		
6 grade	8669 (97.14)	255 (2.86)		
Age (years)	10.32 ± 1.22	10.28 ± 1.24	1.004	0.316
Gender			279.85	<0.001
boy	16752 (94.94)	893 (5.06)		
girl	16645 (98.22)	301 (1.78)		
unknown	151 (96.18)	6 (3.82)		

**Table 2:** Analytical statistics for the association between dyslexia and prenatal and perinatal risk factors.

Variables	Non-dyslexic n (%)	Dyslexic n (%)	Chi-square	*P* value	adjusted *OR* (95% CI)	*P* value
maternal occupation			72.79	<0.001		
professional technical staff	3465 (10.86)	63 (5.71)				
principal of institution and government	977 (3.06)	13 (1.18)				
office staff	1904 (5.97)	35 (3.17)				
business staff	5171 (16.21)	171 (15.5)				
service staff	5987 (18.77)	233 (21.12)				
farmer, forestry, fisher	2203 (6.91)	95 (8.61)				
factory worker	3387 (10.62)	136 (12.33)				
classification of inconvenience	8809 (27.61)	357 (32.37)				
maternal education			156.65	<0.001		
junior high school or below	12498 (38.78)	634 (55.71)				
senior high school or equivalency	13573 (42.12)	405 (35.59)				
junior college	4123 (12.79)	76 (6.68)				
college diploma or above	2032 (6.31)	23 (2.02)				
family income (RMB/month/person)			23.87	<0.001		
≥3000	13732 (48.63)	426 (41.72)				
2000–2999	8722 (30.89)	332 (32.52)				
1000–1999	4680 (16.57)	214 (20.96)				
<1000	1104 (3.91)	49 (4.8)				
Family history of neuropsychiatric diseases^*^	270 (0.8)	25 (2.3)	24.731	<0.001	2.15 (1.30–3.55)	0.003
prenatal factors						
infectious diseases	1852 (5.83)	104 (9.17)	21.914	<0.001	1.59 (1.25–2.03)^a^	<0.001
anti-infectious medication	437 (1.39)	24 (2.18)	4.754	0.029	1.03 (0.93–1.14)^a^	0.548
epilepsy	165 (0.52)	7 (0.62)	0.203	0.652	0.96 (0.62–1.48)^b^	0.845
antiepileptic drugs	103 (0.32)	4 (0.36)	0.037	0.848	1.26 (0.30–5.36)^b^	0.755
hypertension	132 (0.42)	3 (0.26)	0.609	0.435	0.66 (0.34–1.28)^c^	0.220
anti-hypertension medication	88 (0.28)	3 (0.27)	0.003	0.956	1.11 (0.69–1.8)^c^	0.672
diabetes	23 (0.07)	1 (0.09)		0.569	1.91 (0.25–14.63)	0.534
antipsychotics	108 (0.34)	10 (0.91)	9.392	0.002	2.06 (0.89–4.76)	0.089
anemia	863 (2.72)	32 (2.82)	0.047	0.828	1.01 (0.95~1.07)	0.790
smoking	44 (0.20)	4 (0.53)	3.840	0.050	0.42 (0.13–1.38)	0.151
gestational weeks			13.530	<0.001		
full-term (37–42) (ref.)	27110 (88.28)	906 (84.67)			1	
preterm (<37)	2051 (6.68)	89 (8.32)			1.30 (1.01–1.66)	0.040
post-term (>42)	1547 (5.04)	75 (7.01)			1.31 (0.99–1.74)	0.055
perinatal factors						
difficult vaginal delivery	532 (1.75)	33 (3.12)	10.769	0.001	1.58 (1.03–2.42)^d^	0.036
neonatal asphyxia	436 (1.42)	40 (3.76)	38.203	<0.001	2.38 (1.61–3.52)^d^	<0.001
birth injury	143 (0.46)	5 (0.47)	0.001	0.983	0.88 (0.49–1.56)	0.662
neonatal aspiration pneumonia	188 (0.61)	5 (0.47)	0.343	0.558	0.81 (0.56–1.19)	0.293
neonatal jaundice	1058 (3.44)	41 (3.85)	0.519	0.471	1.09 (0.99–1.19)	0.056
neonatal infectious diseases	232 (0.75)	4 (0.38)	2.005	0.157	0.68 (0.46–1.01)	0.052

Note: *family history of neuropsychiatric diseases include epilepsy, mental retardation, ADHD, schizophrenia, depression, Parkinson’s disease, ASD, chorea, Tourette syndrome.

^a^The maternal infectious disease and maternal infectious medicine were mutually adjusted.

^b^The maternal epilepsy and antiepileptic drugs were mutually adjusted.

^c^The maternal hypertension and anti-hypertension medication were mutually adjusted.

^d^The difficult vaginal delivery and neonatal asphyxia were mutually adjusted. All the prenatal and perinatal factors were adjusted for maternal education, maternal occupation, and family income.

## References

[b1] Peterson R. L. & Pennington B. F. Developmental dyslexia. Lancet 379, 1997–2007 (2012). 22513218 10.1016/S0140-6736(12)60198-6 PMC3465717

[b2] Casey R., Levy S. E., Brown K. & Brooks-Gunn J. Impaired emotional health in children with mild reading disability. J. Dev. Behav. Pediatr. 13, 256–260 (1992). 1506463

[b3] Heiervang E., Stevenson J., Lund A. & Hugdahl K. Behaviour problems in children with dyslexia. Nord. J. Psychiatry 55, 251–256 (2001). 11839115 10.1080/080394801681019101

[b4] Maughan B., Rowe R., Loeber R. & Stouthamer-Loeber M. Reading problems and depressed mood. J. Abnorm. Child Psychol. 31, 219–229 (2003). 12735404 10.1023/a:1022534527021

[b5] Willcutt E. G. & Pennington B. F. Psychiatric comorbidity in children and adolescents with reading disability. J. Child Psychol. Psychiatry 41, 1039–1048 (2000). 11099120

[b6] McLaughlin M. J., Speirs K. E. & Shenassa E. D. Reading disability and adult attained education and income: evidence from a 30-year longitudinal study of a population-based sample. J. Learn. Disabil. 47, 374–386 (2014). 22983608 10.1177/0022219412458323

[b7] Demonet J. F., Taylor M. J. & Chaix Y. Developmental dyslexia. Lancet 363, 1451–1460 (2004). 15121410 10.1016/S0140-6736(04)16106-0

[b8] Stevenson H. W. *et al.* Reading disabilities: the case of Chinese, Japanese, and English. Child Dev. 53, 1164–1181 (1982). 7140425

[b9] Chan D. W., Ho C. S. h., Tsang S. m., Lee S. h. & Chung K. K. H. Prevalence, gender ratio and gender differences in reading-related cognitive abilities among Chinese children with dyslexia in Hong Kong. Educ. Stud. 33, 249–265 (2007).

[b10] You H. *et al.* Neural deficits in second language reading: fMRI evidence from Chinese children with English reading impairment. NeuroImage 57, 760–770 (2011). 21146615 10.1016/j.neuroimage.2010.12.003 PMC3499033

[b11] Gong J. *et al.* A functional polymorphism in lnc-LAMC2-1:1 confers risk of colorectal cancer by affecting miRNA binding. Carcinogenesis 37, 443–451 (2016). 26905585 10.1093/carcin/bgw024

[b12] The Wellcome Trust Case Control Consortium. Genome-wide association study of 14,000 cases of seven common diseases and 3,000 shared controls. Nature 447, 661–678 (2007). 17554300 10.1038/nature05911 PMC2719288

[b13] Roeske D. *et al.* First genome-wide association scan on neurophysiological endophenotypes points to trans-regulation effects on SLC2A3 in dyslexic children. Mol. Psychiatry 16, 97–107 (2011). 19786962 10.1038/mp.2009.102

[b14] Shao S. *et al.* The Roles of Genes in the Neuronal Migration and Neurite Outgrowth Network in Developmental Dyslexia: Single- and Multiple-Risk Genetic Variants. Mol. Neurobiol. 53, 3967–3975 (2015). 26184631 10.1007/s12035-015-9334-8

[b15] Friend A., Defries J. C. & Olson R. K. Parental education moderates genetic influences on reading disability. Psychol. Sci. 19, 1124–1130 (2008). 19076484 10.1111/j.1467-9280.2008.02213.x PMC2605635

[b16] Korat O., Arafat S. H., Aram D. & Klein P. Book Reading Mediation, SES, Home Literacy Environment, and Children’s Literacy: Evidence from Arabic-Speaking Families. First Lang. 33, 132–154 (2013).

[b17] Sun Z. *et al.* Prevalence and associated risk factors of dyslexic children in a middle-sized city of China: a cross-sectional study. PloS one 8, doi: 10.1371/journal.pone.0056688 (2013). PMC3574109 23457604

[b18] He Z. *et al.* Does long time spending on the electronic devices affect the reading abilities? A cross-sectional study among Chinese school-aged children. Res. Dev. Disabil. 35, 3645–3654 (2014). 25247847 10.1016/j.ridd.2014.08.037

[b19] Kovachy V. N., Adams J. N., Tamaresis J. S. & Feldman H. M. Reading abilities in school-aged preterm children: a review and meta-analysis. Dev. Med. Child Neurol. 57, 410–419 (2015). 25516105 10.1111/dmcn.12652 PMC4397135

[b20] Harris M. N. *et al.* ADHD and learning disabilities in former late preterm infants: a population-based birth cohort. Pediatrics 132, doi: 10.1542/peds.2012-3588 (2013). PMC3876753 23979091

[b21] Cho K., Frijters J. C., Zhang H., Miller L. L. & Gruen J. R. Prenatal exposure to nicotine and impaired reading performance. J. Pediatr. 162, 713–718 (2013). 23122624 10.1016/j.jpeds.2012.09.041 PMC3577994

[b22] Mascheretti S. *et al.* An assessment of gene-by-environment interactions in developmental dyslexia-related phenotypes. Genes Brain Behav. 12, 47–55 (2013). 23176554 10.1111/gbb.12000

[b23] Whitehouse A. J., Robinson M., Newnham J. P. & Pennell C. E. Do hypertensive diseases of pregnancy disrupt neurocognitive development in offspring? Paediatr. Perinat Epidemiol. 26, 101–108 (2012). 22324495 10.1111/j.1365-3016.2011.01257.x

[b24] van Handel M., Swaab H., de Vries L. S. & Jongmans M. J. Long-term cognitive and behavioral consequences of neonatal encephalopathy following perinatal asphyxia: a review. Eur. J. Pediatr. 166, 645–654 (2007). 17426984 10.1007/s00431-007-0437-8 PMC1914268

[b25] Hokkanen L., Launes J. & Michelsson K. Adult neurobehavioral outcome of hyperbilirubinemia in full term neonates-a 30 year prospective follow-up study. PeerJ. 2, doi: 10.7717/peerj.294 (2014). PMC3961148 24688870

[b26] Rutter M. *et al.* Sex differences in developmental reading disability: new findings from 4 epidemiological studies. Jama 291, 2007–2012 (2004). 15113820 10.1001/jama.291.16.2007

[b27] Evans T. M., Flowers D. L., Napoliello E. M. & Eden G. F. Sex-specific gray matter volume differences in females with developmental dyslexia. Brain Struct. Funct. 219, 1041–1054 (2014). 23625146 10.1007/s00429-013-0552-4 PMC3775969

[b28] Krizman J., Skoe E. & Kraus N. Sex differences in auditory subcortical function. Clin. Neurophysiol. 123, 590–597 (2012). 21855407 10.1016/j.clinph.2011.07.037 PMC3226913

[b29] Oksenberg N., Stevison L., Wall J. D. & Ahituv N. Function and regulation of AUTS2, a gene implicated in autism and human evolution. PLoS Genet. 9, doi: 10.1371/journal.pgen.1003221 (2013). PMC3547868 23349641

[b30] Labouesse M. A., Langhans W. & Meyer U. Long-term pathological consequences of prenatal infection: beyond brain disorders. Am. J. Physiol. Regul. Integr. Comp. Physiol. 309, doi: 10.1152/ajpregu.00087.2015 (2015). 25924881

[b31] Patrich E., Piontkewitz Y., Peretz A., Weiner I. & Attali B. Maternal immune activation produces neonatal excitability defects in offspring hippocampal neurons from pregnant rats treated with poly I:C. Sci. Rep. 6, doi: 10.1038/srep19106 (2016). PMC4705483 26742695

[b32] Choi G. B. *et al.* The maternal interleukin-17a pathway in mice promotes autism-like phenotypes in offspring. Science 351, 933–939 (2016). 26822608 10.1126/science.aad0314 PMC4782964

[b33] Moster D., Lie R. T. & Markestad T. Long-term medical and social consequences of preterm birth. N. Engl. J. Med. 359, 262–273 (2008). 18635431 10.1056/NEJMoa0706475

[b34] Kelly C. E. *et al.* Moderate and late preterm infants exhibit widespread brain white matter microstructure alterations at term-equivalent age relative to term-born controls. Brain Imaging Behav. 10, 41–49 (2016). 25739350 10.1007/s11682-015-9361-0

[b35] Vandermosten M. *et al.* A tractography study in dyslexia: neuroanatomic correlates of orthographic, phonological and speech processing. Brain 135, 935–948 (2012). 22327793 10.1093/brain/awr363

[b36] Wallander J. L. *et al.* Brain research to ameliorate impaired neurodevelopment–home-based intervention trial (BRAIN-HIT). BMC Pediatr. 10, doi: 10.1186/1471-2431-10-27 (2010). PMC2873519 20433740

[b37] Ranasinghe S. *et al.* Reduced Cortical Activity Impairs Development and Plasticity after Neonatal Hypoxia Ischemia. J. Neurosci. 35, 11946–11959 (2015). 26311776 10.1523/JNEUROSCI.2682-14.2015 PMC4549405

[b38] Allen V. M., Baskett T. F., O’Connell C. M., McKeen D. & Allen A. C. Maternal and perinatal outcomes with increasing duration of the second stage of labor. Obstet. Gynecol. 113, 1248–1258 (2009). 19461419 10.1097/AOG.0b013e3181a722d6

[b39] Rosen M. G., Debanne S. M., Thompson K. & Dickinson J. C. Abnormal labor and infant brain damage. Obstet. Gynecol. 80, 961–965 (1992). 1280353

[b40] El Marroun H., White T., Verhulst F. C. & Tiemeier H. Maternal use of antidepressant or anxiolytic medication during pregnancy and childhood neurodevelopmental outcomes: a systematic review. Eur. Child Adolesc. Psychiatry 23, 973–992 (2014). 24863148 10.1007/s00787-014-0558-3

[b41] Wang Zhong *et al.* Reliability and validity of revised pupil rating scale in regional implementation of learning disability screening. Chin. Prev. Med. 11, 682–685 (2010).

